# ANK3 Gene Polymorphism Rs10994336 Influences Executive Functions by Modulating Methylation in Patients With Bipolar Disorder

**DOI:** 10.3389/fnins.2021.682873

**Published:** 2021-08-04

**Authors:** Lili Tang, Juan Liu, Yue Zhu, Jia Duan, Yifan Chen, Yange Wei, Xiaohong Gong, Fei Wang, Yanqing Tang

**Affiliations:** ^1^Department of Psychiatry, The First Affiliated Hospital of China Medical University, Shenyang, China; ^2^Early Intervention Unit, Department of Psychiatry, Affiliated Nanjing Brain Hospital, Nanjing Medical University, Nanjing, China; ^3^State Key Laboratory of Genetic Engineering and Human Phenome Institute, School of Life Sciences, Fudan University, Shanghai, China

**Keywords:** bipolar disorder, executive function, ANK3, methylation, mediation effect

## Abstract

**Background:** A large body of evidence suggests that epigenetic modification including DNA methylation plays a critical role in BD's pathogenesis while the identification of methylation quantitative trait loci (meQTLs) shed light on the interpretation of the function of genetic variants in non-coding regions. The intronic single nucleotide polymorphism (SNP) rs10994336 within the ANK3 has emerged as one of the most replicated risk variants for bipolar disorder (BD) in genome-wide association studies. Whether rs10994336 functions as a meQTL to mediate the association between genotype and phenotype remains unclear.

**Method:** A total of 154 patients with BD and 181 healthy controls (HC) were recruited. The genotypes of rs10994336 and methylation levels of CpG sites within ANK3 were tested. Executive functions were assessed using a computerized version of the Wisconsin Card Sorting Test (WCST).

**Results:** Bipolar disorder patients with the risk-T allele of rs10994336 scored lower on tests of executive function compared to homozygous CC carriers, after controlling for age, gender, and education level. No significant difference was found in HC individuals. The risk-T allele is associated with a lower methylation level of CpG site cg02172182 in HC after multiple corrections and replicated in the BD group in the same direction. Further mediation analysis revealed that the cg02172182 methylation significantly mediated the association between the polymorphism rs10994336 and PE index of WCST in patients with BD.

**Conclusion:** Our study suggests that BD-related genetic variant rs10994336 in ANK3 impacts executive functions by modulating ANK3 methylation, supporting the theory that methylation acts as a mediator between genotype and phenotype.

## Introduction

Bipolar Disorder (BD) is one of the most prevalent mental illnesses characterized by mood swings between episodes of mania or hypomania and depression (McIntyre et al., 2020). Genetic factors play a critical role in BD's etiology (Craddock and Sklar, 2013), supported by the estimated heritability as high as ~80% (McGuffin et al., 2003). Family, twin, and adoption studies show that the lifetime prevalence of BD is ~5–10% among first-degree relatives (Lichtenstein et al., 2009) and ~40–70% among monozygotic twins of patients (Bertelsen et al., 1977; Kieseppä et al., 2004). Executive functions are higher-order cognitive processes that enable an individual to make complex decisions and process goal-oriented thoughts and behavior (Jurado and Rosselli, 2007; Miyake and Friedman, 2012). Executive function deficits have been well-reported in BD (Torres et al., 2007; Hellvin et al., 2012; Samamé et al., 2014; King et al., 2019) and their non-affected first-degree relatives (Bora et al., 2008; Kosger et al., 2015), suggesting that executive function deficits are inheritable features in BD (Frantom et al., 2008; Miskowiak et al., 2017). Considering that executive function is genetically less complicated than the disorder as a whole, it may help to understand the mechanism underlying BD by studying the effect of BD-related genes on executive function.

The T allele of the intronic single nucleotide polymorphism (SNP) rs10994336 within the ANK3 gene are considered a risk allele for BD in genome-wide association studies (GWAS) (Ferreira et al., 2008; Scott et al., 2009; Psychiatric GWAS and Consortium Bipolar Disorder Working Group, 2011; Mühleisen et al., 2014; Stahl et al., 2019). Ankyrin 3 (ANK3) encodes Ankyrin-G, a cytoplasmic adaptor protein primarily located at the axonal initial segments and the nodes of Ranvier (Kordeli et al., 1995). Ankyrin-G plays many roles in neurotransmission, ion channel stabilization, cortical development (Durak et al., 2015), myelination (Ching et al., 1999), and neurogenesis in the adult brain (Paez-Gonzalez et al., 2011). AnkG hemizygous mice generated by the gene trapping approach demonstrated obvious cognitive impairment in an animal model study (Liu et al., 2017) and knockdown of ANK3 in drosophila is associated with memory deficits (Iqbal et al., 2013). Several studies have examined the association between rs10994336 polymorphism and executive functions in BD, a study of 49 patients with BD observed no effect of the rs10994336 genotype on executive function, memory, or general intelligence (Hori et al., 2014) and another study on 47 patients with BD either detected no association between the rs10994336 genotype and executive function (Ruberto et al., 2011). Since BD is a complex disorder and is influenced by a number of genetic variants and environmental exposures, the effect of a single risk allele on phenotype is small, thus, the larger the sample size is, the more power to detect the effect. Considering that the sample size included in those studies were relatively small, further larger sample studies are required to examine whether rs10994336 polymorphism could influence executive functions in BD.

Epigenetic modification including DNA methylation may play a critical role in BD's pathogenesis (Rakyan et al., 2011; Fries et al., 2016; Greenberg and Bourc'his, 2019). Both candidate studies and genome-wide methylation approaches using brain or blood samples have demonstrated promising methylation alterations associated with BD (Gürel et al., 2020). Many risk loci associated with BD are located in non-coding regions of the genome, suggesting that gene regulation plays a role in the disease pathology. Furthermore, different genotypes at a certain locus can alter DNA methylation pattern through allele-specific methylation (Schalkwyk et al., 2010; Bell et al., 2011). These sites are named methylation quantitative trait loci (meQTLs) and have been found throughout the genome in multiple tissues (Gibbs et al., 2010; Schalkwyk et al., 2010; Bell et al., 2011). Interestingly, it has been observed that meQTLs overlap with risk SNPs associated with diseases like BD, schizophrenia (SCZ), and Alzheimer's disease (Gamazon et al., 2013; Gaunt et al., 2016; Hannon et al., 2016). A previous study observed a significant overlap of meQTL between ancestral groups, developmental stages, and tissue types (Smith et al., 2014). Another study also suggests that DNA methylation contains a significant heritable component that is highly stable across the lifespan (Gaunt et al., 2016). These converging results shed light on the interpretation of genetic variants in non-coding regions that the influence of genetic variation on phenotype is potentially mediated through the allele-specific epigenetic processes.

In this study, we sought to study the associations between the rs10994336 variant of ANK3 and executive functions in patients with BD and healthy controls (HCs), and examine whether rs10994336 polymorphism functions as a meQTL and changes the site methylation to mediate the influences of rs10994336 on executive functions.

## Materials and Methods

### Participants

A total of 335 participants between the age of 13 and 60 years were included in this study, including 154 with BD and 181 HCs. All participants with BD were recruited from the inpatient and outpatient service at the Department of Psychiatry, First Affiliated Hospital of China Medical University and Mental Health Center of Shenyang. The recruitment interval was from April 2011 to May 2019. In adult participants (≥18 years old), the Axis I diagnosis (or its absence) were made by two experienced clinical psychiatrists using the Structured Clinical Interview Diagnostic (SCID) and Statistical Manual of Mental Disorder, Fourth Edition (DSM-IV). In participants younger than 18 years, the diagnosis (or absence) of Axis I Disorder were conducted using the Schedule for Affective Disorder and Schizophrenia for School-Age-Children-present (K-SADS-PL) by two experienced child psychiatrists. All the participants with BD in this study met the DSM-IV diagnostic criteria for BD and had no other Axis I Disorder. HC participants were recruited from Shenyang, China and surrounding cities by publicly posted advertisement. All HC participants had no current or lifetime history of an Axis I disorder or a history of psychotic, mood, or other Axis I Disorder in first-degree relatives, as determined from a detailed family history. Participants were excluded if they met any of the following criteria: (1) any history of major medical disease; (2) any history of moderate or severe head injury, head trauma, neurological disorder, or mental retardation; (3) lifetime substance/alcohol abuse or dependence; and/or (4) the presence of a concurrent and major physical illness that could lead to mood disorder symptoms. The current study was approved by the Ethics Committee of the First Affiliated Hospital of China Medical University (Shenyang China). Each participant provided a written informed consent after a complete description of the study. If their age were <18 years, they, as well as their parental/legal guardian, provided a written informed consent.

### Genotyping

Venous blood samples were collected from the participants for DNA extraction pursuant to the standard procedures. Genotype information of rs10994336 was extracted from the results of the Illumina Global Screening Array-24 v1.0 BeadChip. Participants were further divided into two groups: a CC group (CC genotype; 91 BD, 99 HC; mean age = 27.42 ± 9.70 years, 34.74% female) and risk T-carrier group (TT/CT genotypes; 63 BD, 82 HC; mean age = 27.28 ± 9.69 years, 33.56% female). Genotype frequencies were consistent with Hardy-Weinberg equilibrium (HWE) expectations.

### DNA Methylation Data of ANK3

DNA methylation level was assessed using the Infinium Human Methylation850 (850K) Beadchip array (Moran et al., 2016). To avoid batch effect, samples were processed in random order. All samples passed the quality assessment of assay performance requirements implemented in the Genome Studio software integrated controls dashboard. All DNA methylation levels were expressed as β-values, ranging from 0 to 1, calculated as M/(M+U), where M is the signal from methylated beads and U is the signal from unmethylated beads at the targeted CpG site and corrected for cell heterogeneity using the R package ChAMP (Tian et al., 2017). The genome coordinates provided by Illuminia (GRCh37/hg19) were used to map the independent methylation CpG site to a certain gene. Then, the β-values of all CpG sites within ANK3 were extracted for further analysis.

### Cognitive Measures

The executive function was measured in 297 participants including 133 BD and 164 HC by the computerized version of the Wisconsin Card Sorting Test (WCST) (Heaton, 1981; Heaton et al., 1993), the most widely employed neuropsychological test for the assessment of executive function. This test requires subjects to alter response strategies and use previous irrelevant information to solve a problem. The WCST provides five indices as follows: correct response (CR), completed categories (CC), total errors (TE), perseverative errors (PE), and non-perseverative errors (NPE).

### Statistical Analyses

#### Demographic and Clinical Data

Independent two-sample *t*-tests or the chi-square tests were employed to investigate differences in the demographic and clinical data between the TT/TC group and CC group in participants with BD or HC participants. Continuous variables were presented as mean ± *SD*. The threshold for significance level was defined as *P* < 0.05.

#### Association Between Rs10994336 and Cognition

For all the participants who completed the WCST measures, we conducted one-way ANOVA with age, gender, and education years as covariates to explore the effect of genotype on the five indices in WCST. Statistical significance was set at two-tailed *P* < 0.05.

#### Association Between Rs10994336 and ANK3 Methylation Levels

A total of 160 CpG sites of ANK3 were included and all the corresponding β-values were calculated for each individual. The association between the TT/TC and CC groups of rs10994336 and each methylation level of the 160 CpG sites was tested by a linear regression model adjusted with age and gender as covariates in HC participants and medication factor added as an extra covariate in the BD group. The significance level for the association analyses was set at *p* < 0.05. A Benjamin–Hochberg false-discovery rate-corrected (FDR) correction was used to avoid type I error.

#### Mediation Analysis

To test whether the effect of rs10994336 genotype on cognition was potentially mediated by the methylation level of ANK3, we conducted mediation analysis on PE index using the PROCESS macro for SPSS, with a 5,000 bias-corrected bootstrap sample for significance testing. The mediation analysis was comprised of three steps of regression. In the first step, the outcome variable (PE index of WCST) was regressed on the independent variable (rs10994336 genotypes), which indicates the total effect of the predictor on the outcome variable. In the second step, the mediator variable (cg02172182 methylation) was regressed on the independent variable (rs10994336 genotypes), and in the final step, the outcome variable (PE index of WCST) was regressed on the mediator (cg02172182 methylation) and the independent variable (rs10994336 genotypes). An indirect or mediating effect is supported when the significant association between the independent variable (rs10994336 genotypes) and outcome variable (PE index of WCST) diminishes after introducing a third variable, the mediator. In addition, age and gender were included as covariates in all mediation models and the study variables were standardized.

All the statistics analyses were conducted using the SPSS software except the association analysis between rs10994336 genotypes and methylation level of ANK3 which was performed by the R statistical package (http://cran.r-project.org) for multiple repeated tests.

## Results

### Demographic and Clinical Characteristics

Demographic characteristics and clinical variables of patients with BD and HC subjects are presented in Table 1. There were no significant differences in age, gender, education level, or medication use between the CC and TT/TC groups in BD or HC participants.

**Table 1:** Demographic and clinical characteristics of different allele group in the BD and HC groups.

	**BD (** ***n*** **=** **154)**		**HC (** ***n*** **=** **181)**		**P1**	**P2**	**P3**
	**TT/TC**	**CC**	**TT/TC**	**CC**			
	**(*n* = 63)**	**(*n* = 91)**	**(*n* = 82)**	**(*n* = 99)**			
Gender (M/F)	19/44	28/64	30/52	39/60	0.971	0.699	0.134
Age (years)	25.67 ± 10.56	26.85 ± 9.67	28.51 ± 8.77	27.95 ± 9.69	0.478	0.687	0.084
Education (years)	12.33 ± 3.19	12.7 ± 3.36	14.74 ± 3.3	14.55 ± 3.63	0.502	0.725	0.131
Age at onset (years)	23.5 ± 10.2	23.89 ± 9.21	NA	NA	0.818	NA	NA
Medication (Y/N)	40/23	61/30	NA	NA	0.649	NA	NA

*BD, Bipolar Disorder; HC, Health control; M/F, male/female; Y/N, yes/no; NA, not applicable. P1, comparison between TT/TC group and CC group in BD; P2, comparison between TT/TC group and CC group in HC; P3, comparison between BD and HC*.

### Effects of Rs10994336 on Cognition

**Figure 1:**
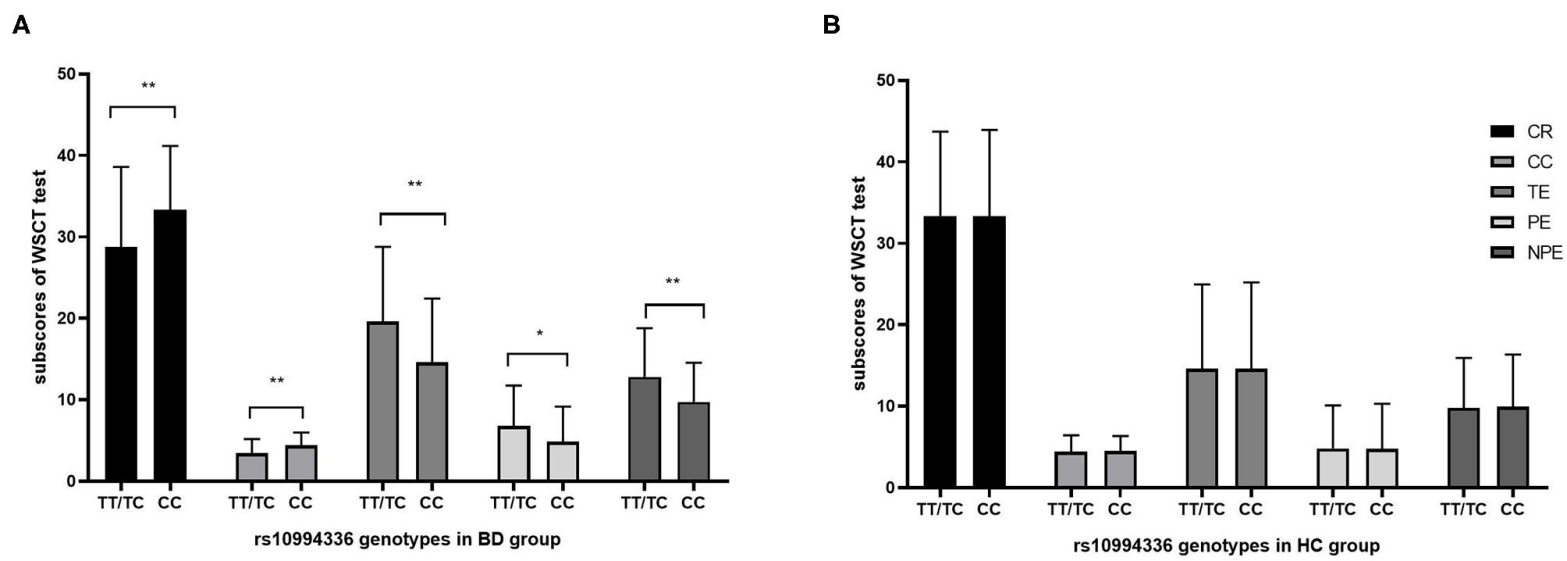
The association between genotypes of rs10994336 and cognition. Comparisons were made for five indices of WSCT between rs10994336 T-allele carriers and C-allele homozygotes in patients with BD **(A)** and in HC **(B)**. WCST, Wisconsin Card Sorting Test; BD, bipolar disorder; HC, healthy control; CR, correct response; CC, completed categories; TE, total errors; PE, perseverative errors; NPE, non-perseverative errors. Error bars represent standard errors of the means. *P < 0.05, **P < 0.01 (according to the ANCOVA controlling for age, gender, and education).

The one-way ANOVA analysis was performed to investigate the effect of rs10994336 on cognition. The results were shown in Figure 1A for participants with BD and Figure 1B for HC participants, in which all five indices of WCST were significantly different between the T-allele carriers and homozygous CC individuals in BD, while no significant difference was detected in HC.

### Effects of Rs10994336 on ANK3 Methylation Levels

**Figure 2:**
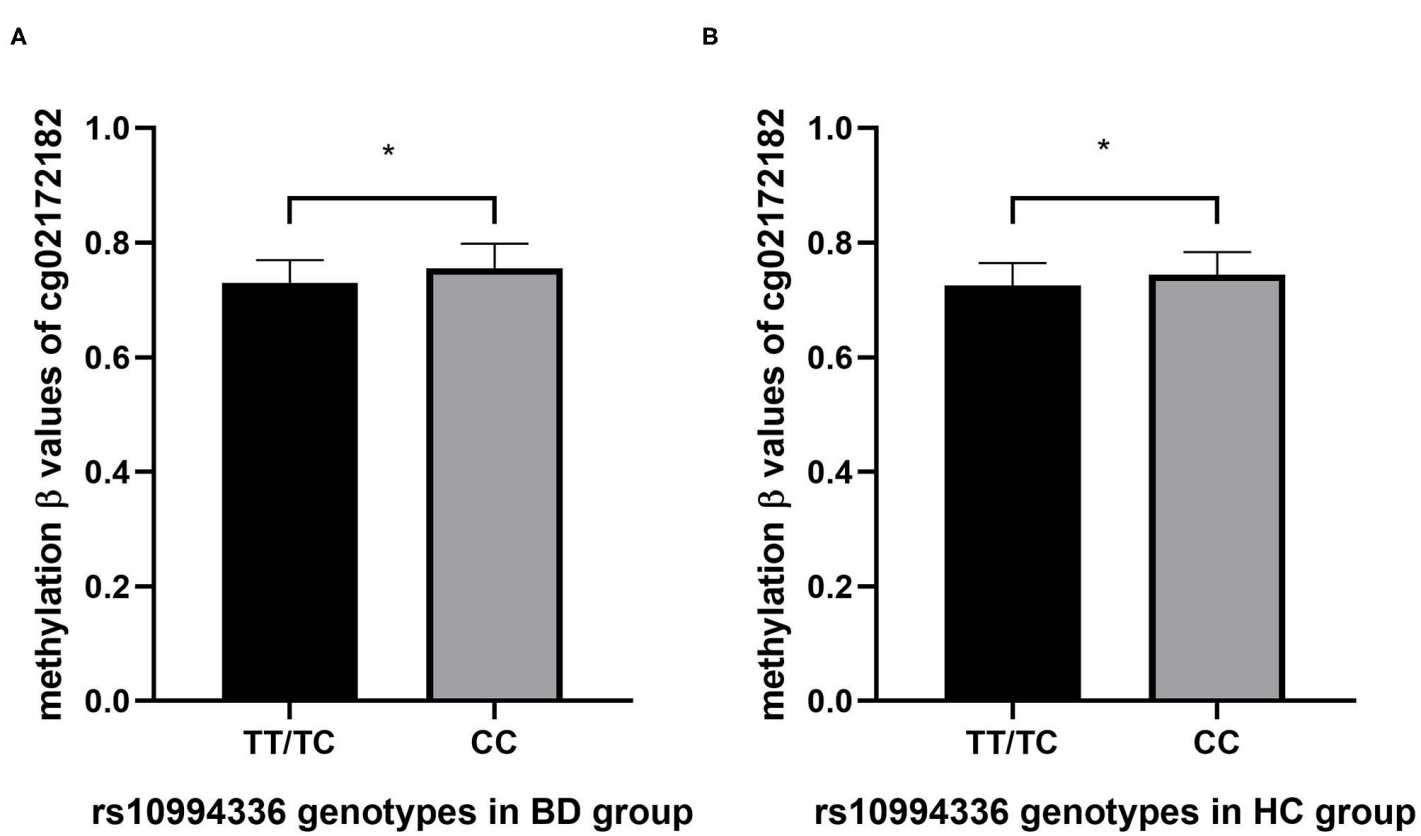
The association between genotypes of rs10994336 and the methylation level at CpG site cg2172182 within ANK3 in patients with BD **(A)** and in HC **(B)**. BD, bipolar disorder; HC, healthy control. *P < 0.05 after adjusting for multiple tests by the Benjamini-Hochberg false-discovery rate-corrected (FDR) correction.

We conducted an association study between rs10994336 and the methylation levels of ANK3 (160 CpG sites) in BD and HC, respectively. After correcting for multiple tests, two CpG sites (cg02172182, P 0.017; cg093110194, P 0.011) showed a significant association with the genotype in BD participants, while eight CpG sites (cg05852740, P 0.003; cg06072426, P 0.1; cg11886031, P 0.045; cg12318342, P 0.034; cg02172182, P 0.047; cg 21429687 0.044; cg26504362, P 0.044; cg01186212, P 0.047) were associated significantly with the genotypes of rs10994336 in HC participants. The results of the association between the genotypes of rs10994336 and methylation level of CpG site cg02172182 were consistent in patients with BD (Figure 2A) and in HC (Figure 2B) individuals, where the TT/TC group had a lower methylation level of cg02172182 both in BD and HC.

### Mediation Effect

**Figure 3:**
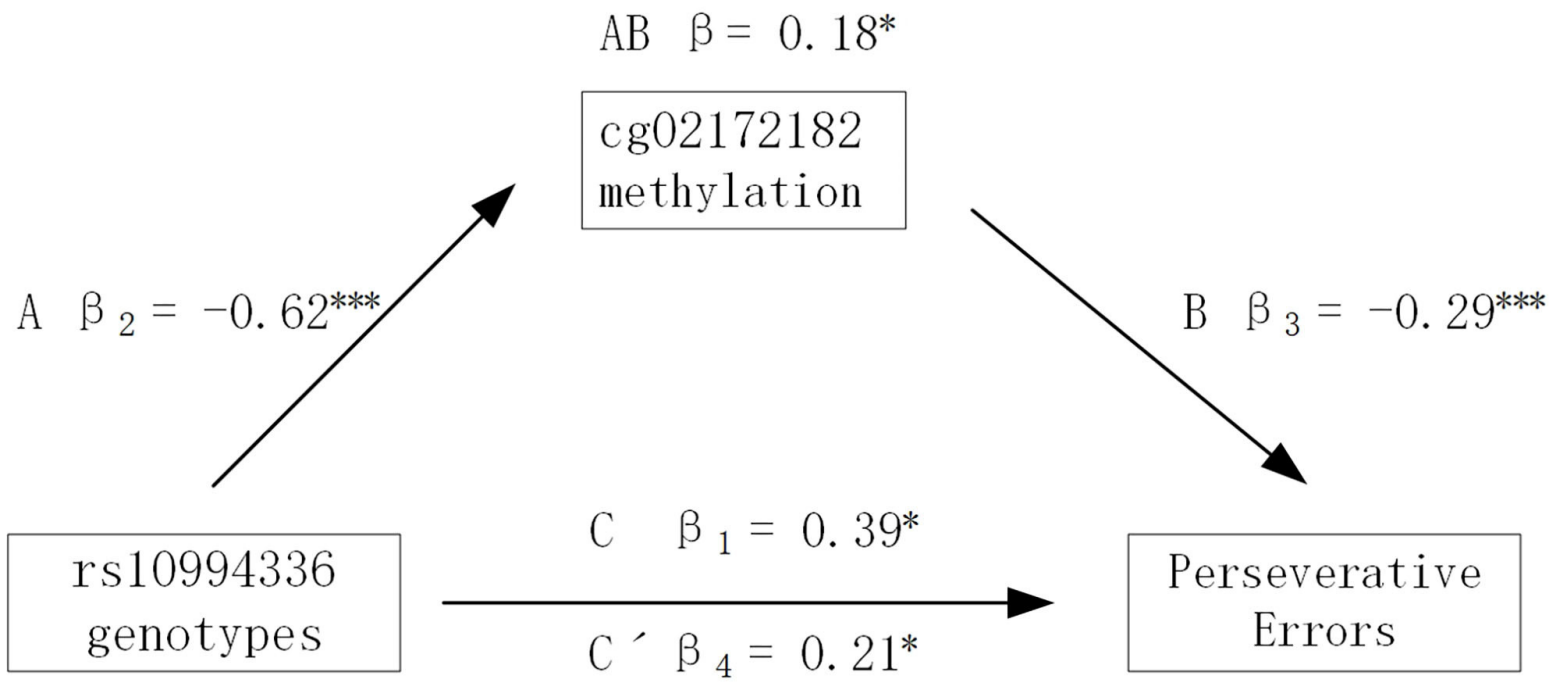
CpG site cg02172182 methylation mediation. Summary of an analysis of the mediating effect of cg02172128 methylation on the relationship between rs10994336 polymorphism and Perseverative Errors (PE) indices of the Wisconsin Card Sorting Test (WCST). Path C represents a total effect of rs10994336 genotype on PE, path A represents the effect of rs10994336 genotype on cg02172182 methylation, path B represents the effect of cg02172182 methylation on PE, and path C' represents the direct effect of rs10994336 polymorphism on PE after taking into account cg02172128 methylation as a mediator. *P < 0.05, **P < 0.01, ***P < 0.001. Path AB is the mediation (indirect) effect and is significant at *P* < 0.05 based on confidence intervals from bias-corrected bootstrapping of 5,000 samples.

Given the significant effect of polymorphism rs10994336 on executive functions in the BD group and that rs10994336 was detected to be a meQTL by influencing the methylation level of CpG site cg02172182 both in the HC and BD groups, we assessed whether the methylation level of cg02172182 mediated the association between rs10994336 and executive functions. The results showed that the methylation level of cg02172182 had a significant mediation effect on the association between ANK3 rs10994336 genotypes and PE index of WCST in the BD group (Figure 3). In step one of the mediation analysis, rs10994336 genotypes significantly predicted the PE index of WCST (path C), and rs10994336 genotypes also predicted cg02172182 methylation (path A) in step two. In the final step, we found that the predictive effect of rs10994336 genotypes on PE (index of WCST) became less significant after introducing cg02172182 methylation as a mediator (path C' < path C), thus demonstrating that cg02172182 methylation partially mediated the association between rs10994336 genotypes and PE index of WCST.

## Discussion

In the present study, we found that the risk T-allele carriers of rs10994336 showed a worse WCST performance compared to those with homozygous CC genotype in the BD group. Moreover, rs10994336 affects the methylation level of specific CpG site within ANK3 as a meQTL in the BD and HC groups, which mediates the effect of rs10994336 on executive functions. To the best of our knowledge, it is the first study to document the mediation effect of ANK3 methylation between BD-related genetic variant rs10994336 within ANK3 and executive functions in BD.

Our results suggest that the risk allele of rs10994336 of ANK3 is associated with a poor executive performance in patients with BD after adjusting for age, gender, and education. This is consistent with the result of a previous study conducted among 173 patients with first-episode psychosis showing that the polymorphism rs1938526 of ANK3 gene, which was in high linkage disequilibrium with rs10994336, was associated with cognitive domains of reasoning and problem solving, though at a marginal significance (Cassidy et al., 2014). Moreover, the loss of association between rs10994336 and executive functions in the HC group was consistent with two previous studies which detected no effect of this SNP on measures of executive function either in HC individuals (Hori et al., 2014) or in healthy males (Roussos et al., 2011). However, two studies on BD failed to examine the association between rs10994336 and executive function, memory, or general intelligence (Ruberto et al., 2011; Hori et al., 2014). It should be noted that the sample size included in those studies were relatively small which reduced the statistical power to test the association. Considering the complex interplay between genetic and environmental factors in the etiology contributing to BD, we speculate that the association between rs10994336 and executive function is predominant in patients with BD, who have a predisposition to severe cognitive deficits due to the complex pathophysiological processes induced by genetic factors. Studies with larger samples are needed to test whether these findings can be replicated.

The polymorphism rs10994336 is located in the intron of ANK3 gene, and its function was unclear until now. We tested whether this SNP affected the methylation level of ANK3. Considering variables such as age, gender, and medication use were believed to influence DNA methylation (Burghardt et al., 2020; Gardea-Resendez et al., 2020; Webb et al., 2020), we add these confounding factors in our linear regression model as covariates to explore the allele-associated methylation. After adjusting these confounders, the effect of rs10994336 polymorphism on methylation of the CpG site cg02172182 within the gene body of ANK3 was observed in HC and replicated in patients with BD in the same direction where the risk T-allele carriers presented a reduced methylation relative to homozygous CC participants, suggesting that the effect of genotype on methylation is not driven by the disease and rs10994336 could be referred to as a reliable meQTL. Cg02172182 locates at the gene body region of ANK3 and ~1.5 kb upstream of rs10994336, which functions by cis-acting meQTL that can act over a range of distances, from a few base pairs to distances of over 500 kb. Our mediation analysis model in BD supports the theory that methylation acts as a mediator between genotype and phenotype, as the effect of rs10994336 polymorphism on PE was significantly mediated by the methylation of cg02172182. Although the methylation patterns are highly heterogeneous between different tissue types and at different life stages, genetic effects on methylation are found to be rather stable across life course and different tissue types (Smith et al., 2014; Gaunt et al., 2016). A previous study identified separately and compared genome-wide meQTLs from brain prefrontal cortex, whole blood, and saliva and observed significant overlap of meQTLs among tissues (Smith et al., 2014). Moreover, another study also implicated that the DNA methylation status of many CpG sites in the brain were mirrored in the blood and that common meQTLs were also detected between these two tissues (Gaunt et al., 2016). These converging findings support the theory that blood tissue could be used as a surrogate tissue to explore disease relevant processes within the brain. In addition, multiple strands of evidence indicate that DNA methylation plays an important role in several neurobiological processes including neurogenesis (Costello, 2003), neural plasticity (Dulac, 2010), and the formation and maintenance of memories (Halder et al., 2016), which in turn impacts on executive function. Prompted by these findings, it is valid to speculate that the function of rs10994336 as a meQTL in whole blood tissue may mirror the function in brain tissues which influences the methylation of ANK3 gene and further results in executive function impairment in patients with BD.

There are several limitations to the present study. First, the sample size of our study did not have sufficient power to detect the difference of frequencies of rs10994336, or the difference of methylation of ANK3 between BD and HC. Since rs10994336 is a well-established BD risk variant, the difference of genotype or allele frequencies between BD and control will lead to the difference of methylation of this gene, which will have an effect on brain function. This may explain why we only detected the association of rs10994336 with executive functions in BD. Second, the actual effect of rs10994336 polymorphism on ANK3 methylation in brain tissues in which the pathological processes for psychiatric disorder are likely involved could not be directly observed in our study, since brain tissues are not readily accessible in living patients. However, research findings indicate a common overlap of meQTLs between different tissue types including blood and brain tissues. Third, we did not stratify our samples according to those confounding factors such as age, medication use, and disease status (manic, depressive, and remission) which could affect DNA methylation considering that our sample size was not large enough. However, age and medication use were adjusted in our linear regression models and the effect of rs10994336 polymorphism on cg121722182 methylation remained significant. Future studies with larger samples could further exclude those confounders. Finally, a potential limitation is that the precise mechanism by which methylation of cg121722182 influences executive functions is unclear since it locates at the gene body of ANK3 while the role of methylation in gene bodies is not well-established, but some evidence indicates that methylation in gene bodies is associated with the activation of genes in contrast to the methylation observed in promoter regions which are generally considered to be involved in the content of silence of genes (Greenberg and Bourc'his, 2019). Future studies are needed to address this question.

In summary, using executive functions as a highly heritable endophenotype and combining genetic and epigenetic data, we found that allelic variation of rs10994336 in ANK3 impacts executive functions by moderating methylation, supporting the theory that methylation acts as a mediator between genotype and phenotype. This provides insights into the mechanism of how intron risk variants associated with BD contribute to disease susceptibility.

## Data Availability Statement

The raw data supporting the conclusions of this article will be made available by the authors, without undue reservation.

## Ethics Statement

The studies involving human participants were reviewed and approved by Ethics Committee of First Affiliated Hospital of China Medical University. Written informed consent to participate in this study was provided by the participants' legal guardian/next of kin.

## Author Contributions

YT and FW contributed to the conception and design of the study and provided the technical support. LT performed the data analyses and wrote the manuscript. LT, JL, YW, YZ, JD, and YC contributed to acquisition of data and manuscript preparation. XG helped perform the analysis with constructive discussions. All authors contributed to the article and approved the submitted version.

## Conflict of Interest

The authors declare that the research was conducted in the absence of any commercial or financial relationships that could be construed as a potential conflict of interest.

## Publisher's Note

All claims expressed in this article are solely those of the authors and do not necessarily represent those of their affiliated organizations, or those of the publisher, the editors and the reviewers. Any product that may be evaluated in this article, or claim that may be made by its manufacturer, is not guaranteed or endorsed by the publisher.
